# Demographic and clinical characteristics associated with anxiety and depressive symptom outcomes in users of a digital mental health intervention incorporating a relational agent

**DOI:** 10.1186/s12888-024-05532-6

**Published:** 2024-01-30

**Authors:** Emil Chiauzzi, Andre Williams, Timothy Y. Mariano, Sarah Pajarito, Athena Robinson, Andrew Kirvin-Quamme, Valerie Forman-Hoffman

**Affiliations:** 1 Woebot Health, 535 Mission Street, 14th Floor, San Francisco, CA 94105 USA; 2 RR&D Center for Neurorestoration and Neurotechnology, VA Providence Healthcare System, Providence, RI USA; 3 Department of Psychiatry and Human Behavior, Warren Alpert Medical School of Brown University, Providence, RI USA

**Keywords:** Digital mental health intervention, Chatbot, Conversational agent, Relational agent, Depression, Anxiety, User characteristics, Natural language processing, Artificial intelligence

## Abstract

**Background:**

Digital mental health interventions (DMHIs) may reduce treatment access issues for those experiencing depressive and/or anxiety symptoms. DMHIs that incorporate *relational agents* may offer unique ways to engage and respond to users and to potentially help reduce provider burden. This study tested Woebot for Mood & Anxiety (W-MA-02), a DMHI that employs *Woebot*, a relational agent that incorporates elements of several evidence-based psychotherapies, among those with baseline clinical levels of depressive or anxiety symptoms. Changes in self-reported depressive and anxiety symptoms over 8 weeks were measured, along with the association between each of these outcomes and demographic and clinical characteristics.

**Methods:**

This exploratory, single-arm, 8-week study of 256 adults yielded non-mutually exclusive subsamples with either clinical levels of depressive or anxiety symptoms at baseline. Week 8 Patient Health Questionnaire-8 (PHQ-8) changes were measured in the depressive subsample (PHQ-8 ≥ 10). Week 8 Generalized Anxiety Disorder-7 (GAD-7) changes were measured in the anxiety subsample (GAD-7 ≥ 10). Demographic and clinical characteristics were examined in association with symptom changes via bivariate and multiple regression models adjusted for W-MA-02 utilization. Characteristics included age, sex at birth, race/ethnicity, marital status, education, sexual orientation, employment status, health insurance, baseline levels of depressive and anxiety symptoms, and concurrent psychotherapeutic or psychotropic medication treatments during the study.

**Results:**

Both the depressive and anxiety subsamples were predominantly female, educated, non-Hispanic white, and averaged 38 and 37 years of age, respectively. The depressive subsample had significant reductions in depressive symptoms at Week 8 (mean change =—7.28, SD = 5.91, Cohen’s d = -1.23, *p* < 0.01); the anxiety subsample had significant reductions in anxiety symptoms at Week 8 (mean change = -7.45, SD = 5.99, Cohen’s d = -1.24, *p* < 0.01). No significant associations were found between sex at birth, age, employment status, educational background and Week 8 symptom changes. Significant associations between depressive and anxiety symptom outcomes and sexual orientation, marital status, concurrent mental health treatment, and baseline symptom severity were found.

**Conclusions:**

The present study suggests early promise for W-MA-02 as an intervention for depression and/or anxiety symptoms. Although exploratory in nature, this study revealed potential user characteristics associated with outcomes that can be investigated in future studies.

**Trial Registration:**

This study was retrospectively registered on ClinicalTrials.gov (#NCT05672745) on January 5th, 2023.

**Supplementary Information:**

The online version contains supplementary material available at 10.1186/s12888-024-05532-6.

## Background

Mental health issues have consistently contributed to the worldwide disease burden [1]. This held true before but was exacerbated by the COVID-19 pandemic, given its associated increase of 25% in depressive and anxiety disorders [2]. Stressing already limited mental health treatment access and provider capacity, the increased prevalence has left even more individuals without needed care [3, 4]. In order to mitigate such prolific access and capacity issues, people experiencing symptoms of depression and anxiety may seek interventions with alternative delivery modalities [5]. Indeed, the heightened need for treatment access has led to an increase in the use and study of technology-delivered mental health interventions [6]. Such interventions, known as digital mental health interventions (DMHIs), offer self-guided (no human support), guided (human support specific to the DMHI offered via telephone, video, or text), or blended (a combination of DMHI plus human support) approaches to deliver treatment modules and skill-building interventions [7]. DMHIs have included therapeutic techniques from one or more evidence-based psychotherapies, such as cognitive behavioral therapy (CBT) [7]. In aggregate, DMHIs have been shown to produce at least moderate improvements in depressive and anxiety symptoms [8–10].

Among DMHIs, there has been increased attention on and proliferation of mental health *chatbots* [11, 12] also known as *conversational agents* [13, 14]. Conversational agent-delivered interventions are often available on a smartphone application (app), enabling users access at any time for support and to address immediate problems contextually in real time. Like the broader DMHI category, conversational agent-delivered interventions may be founded in evidence-based psychotherapeutic approaches such as CBT, Acceptance and Commitment Therapy (ACT), and Dialectical Behavior Therapy (DBT) [7, 15–17]. In addition to thoughtful conversational design, some conversational agents are bolstered by artificial intelligence and/or natural language processing (NLP) [18], which may enable response by delivery of therapeutic intervention and psychoeducational content. Conversational agent-delivered interventions may be particularly useful for those who wish to receive help without speaking to a clinician due to stigma or cost concerns [13]. Recent meta-analyses of conversational agent delivered DMHIs indicate that they are generally viewed favorably by users and may enhance access to mental health care and improve mental health outcomes [11, 13, 19].

When conversational agents are built to form an alliance with users over time, they may be referred to as *relational agents* [20, 21]. Some data have demonstrated that relational agents are able to form a working therapeutic alliance with users [22], which has been linked to improved mental health outcomes [23]. These capabilities, coupled with their immediate availability in someone’s moment of need, offer the potential for relational agents to extend the current menu of mental health services available to those in need. Unfortunately, little is known about the characteristics of users who may benefit from relational agent-delivered DMHI [11].

The broader, non-relational agent-specific DMHI literature provides some clues about what types of user demographic or clinical characteristics might be associated with outcomes among relational agent users, but much of the evidence has been inconclusive. DMHI studies of depression and anxiety have reported inconsistent findings in outcomes by characteristics such as gender, age, marital status, or education [24–29]. Although there is preliminary evidence of the feasibility and acceptability of DMHI interventions for racial/ethnic, LGBTQ (lesbian, gay, bisexual, transgender, queer or questioning), and other marginalized or underserved groups, these demographic and self-identity characteristics have been underexplored as predictors of outcomes in the DMHI literature [30, 31]. Individual DMHI studies addressing racial and ethnic minorities have shown promise in reducing depressive symptoms, but large scale prospective studies are lacking [31].

With regard to clinical characteristics, non-relational agent-specific DMHI studies have generally found that users with higher severity levels of anxiety and depression tend to achieve greater improvement in these symptoms than those with lesser severity [8, 10, 32], likely an artifact that these users have more room for improvement [8]. Surprisingly, the association between concurrent mental health treatment (i.e., psychotherapy and/or psychotropic medications) and DMHI-related changes in depression and anxiety symptoms has seen little investigation. One study of health care workers during the COVID-19 pandemic found that exposure to a mobile phone–based mental health intervention did not significantly affect Depression Anxiety Stress Scale-21 (DASS-21) scores in the overall sample, but a subgroup analysis of those receiving psychotherapy or psychotropic medications found significant improvements in this group [33]. While some data exist on demographic and clinical characteristic associations with mental health outcomes among the broad category of DMHIs, the authors could find no such data in the literature specific to relational or conversational agent-delivered DMHIs. Thus, further exploration of putative demographic and clinical characteristics among users of a relational agent-delivered DMHI is needed to illuminate optimal uses as well as perhaps tailor interventions to the unique needs of individuals experiencing anxiety and depressive symptoms.

This present study evaluated Woebot for Mood and Anxiety (W-MA-02), an intervention delivered via *Woebot*, a relational agent that utilizes thoughtful conversational design and some NLP to incorporate elements of  CBT, Interpersonal Psychotherapy (IPT), and DBT into a text-based interface on a smartphone app. Feasibility, acceptability, and efficacy studies using Woebot-based interventions have shown promise in young adults reporting depressive symptoms [34], adults with substance abuse issues [35], adolescents with depressive and/or anxiety symptoms [36], and adult women with postpartum mood concerns [37, 38]. The present study sought to address gaps in the literature specific to a relational agent-delivered DMHI by first measuring depressive or anxiety symptom changes after 8 weeks of W-MA-02 use among participants with self-reported clinical levels of depressive or anxiety symptoms at baseline, respectively. In addition, the study explored associations between participant demographic and clinical characteristics and depressive and anxiety symptom outcomes. The research questions in this exploratory study included the following:

1. Do participants with clinically elevated depressive symptoms at baseline have significant decreases in symptoms at Week 8 of a relational agent-delivered DMHI?
2. Do participants with clinically elevated anxiety symptoms at baseline have significant decreases in symptoms at Week 8 of a relational agent-delivered DMHI?
3. Does the amount of depressive and anxiety symptom reduction at Week 8 of a relational agent-delivered DMHI differ by demographic characteristics, clinical characteristics (i.e., baseline anxiety and depression symptom severity), and involvement in concurrent mental health treatment?

## Methods

### Overall study description

This exploratory study was non-randomized, single-armed, and open-labeled. Study procedures were approved by the WIRB-Copernicus IRB Group (WCG) institutional review board on January 20th, 2022. All participants provided informed consent. Data was collected from W-MA-02 participants between May 11th, 2022 and July 20th, 2022. The study was retrospectively registered on ClinicalTrials.gov (#NCT05672745) on January 5th, 2023.

### Recruitment

A convenience sample was recruited via social media advertisements, such as Facebook and Instagram, containing an embedded hyperlink to the study’s electronic consent form. Upon signing the consent form, study staff emailed participants a link to complete the self-report screening (assessing inclusion and exclusion criteria) and baseline survey questions. The inclusion criteria were: (1) 18 years of age or older, (2) residence in the United States (U.S.), (3) reported English literacy, (4) availability to complete study activities, and (5) ownership or regular access to a smartphone with WiFi and sufficient data access for the duration of the study. The exclusion criteria were: (1) current suicidal ideation with a plan or intent to act or a suicide attempt within the past 12 months, (2) a reported lifetime diagnosis of a psychotic disorder (including schizophrenia or schizoaffective disorder) or bipolar disorder, or (3) previous use of a Woebot application.

Participants who met inclusion and exclusion criteria and who completed the baseline survey assessments within 7 days of receipt were immediately provided with a link to access and download W-MA-02. They were instructed to complete registration within 3 days using the study’s referral code and the same credentials entered at consent. Those who failed to register within 3 days became ineligible for the study and thus were not enrolled. Participants who did not meet eligibility criteria were thanked for their interest, given a list of national crisis resources, and exited from the study.

### Study procedures

**Fig. 1:**
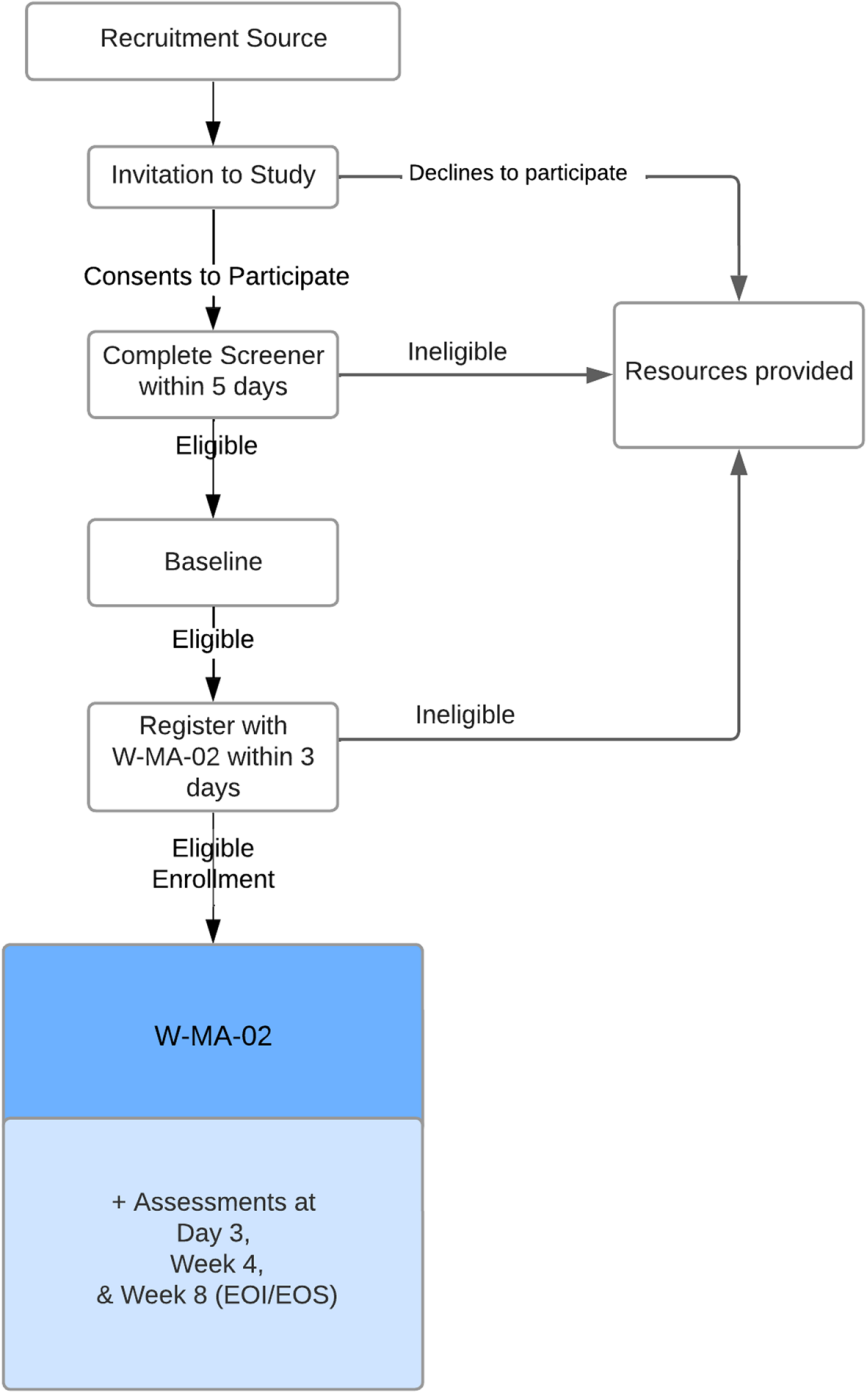
Participant Flow Through Study. EOI = end of intervention. EOS = end of study

Enrolled participants entered the 8-week intervention period, where they were invited to engage with W-MA-02 daily. Participants completed survey assessments that were delivered outside of the application via email invitation at Day 3, Week 4 (mid-intervention), and Week 8 (end of intervention; EOI). Upon the completion of the final survey, participants exited the study and their access to W-MA-02 was discontinued. Participants received a maximum of $100 in Amazon gift cards for completing study assessments ($20, $30, and $50 for Day 3, Week 4, and Week 8, respectively; payments were not linked to app usage). This study reports data collected at baseline and Week 8 (EOI). Figure 1 describes the participant flow through the study.

### W-MA-02 intervention

**Fig. 2:**
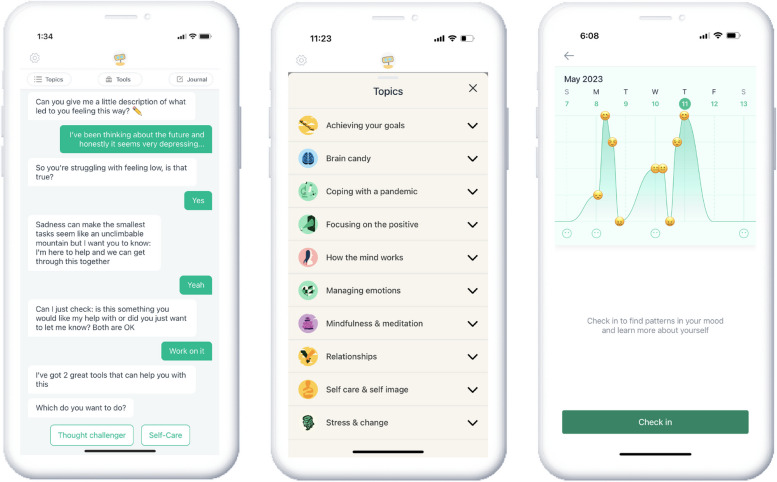
Screenshots of Mood Monitoring within W-MA-02

W-MA-02 is intended for users seeking help with mood management and tracking; it is not mental health disorder-specific. The application is accessible as an iOS or Android smartphone application and hosts the relational agent *Woebot* to guide users through psychotherapeutic content delivered via text-based messages. Using proprietary natural language processing, the user experience centers around goal-oriented conversations based on self-reported real-time needs. These conversations are tailored to help the user develop emotion regulation skills in the context of problems experienced in their everyday lives. W-MA-02 is grounded in scientifically validated psychotherapies (e.g., CBT, IPT, DBT) and helps users address mood monitoring and management, as well as utilizes tools such as progress reflection, gratitude journaling, and mindfulness practice. Previous studies of various Woebot applications, including the W-MA-02 intervention, have provided evidence of feasibility, acceptability and efficacy [34, 35, 38, 39], as well as demonstrated its ability to form a working alliance with users [22]. Sample screenshots from W-MA-02 are shown in Fig. 2.

### Ethical considerations

W-MA-02 is not a crisis service. However, it is equipped with an NLP algorithm that detects patterns in user free-text input that may suggest potential concerning language. Upon detection, W-MA-02 asks the user to confirm if they are in crisis. For all confirmations, W-MA-02 provides a list of resources that includes emergency contact phone numbers and suicide crisis hotline information. W-MA-02 then offers tools to assist with addressing upsetting emotions and thoughts in the moment. The list of resources is also immediately available at any time to the user either from the settings menu or by entering “SOS” in free text. Potential concerning language detection triggers and confirmations were not actively monitored by staff during the study.

Due to the low-risk profile of this study, adverse events (AEs) were assessed by spontaneous participant reports communicated outside of W-MA-02 and followed a predetermined safety protocol. In the event of a spontaneously reported AE to any study staff, the Principal Investigator would be immediately alerted with all available clinical and other details of the event. Based on this initial report, appropriate steps would be taken to gather further information as needed and address the AE. During this study, no such AEs were reported.

### Assessments

Participants were emailed links to survey assessments through the Castor Electronic Data Capture (EDC) [40] platform, with a 7-day window to complete the baseline and Week 8 surveys reported herein. Completion of the baseline survey was considered Day 1 of the intervention period. We tracked the assessment completion rate, defined as completion of both the PHQ-8 and GAD-7 at baseline and Week 8.

### Demographic battery

The baseline survey assessed demographic characteristics, including age, race/ethnicity, marital status, education, sexual orientation, employment status, and health insurance. Gender identity, while collected, did not yield enough individuals who identified outside of the most common categories (Man or Woman) to allow for inclusion in the models. Instead, we included sex assigned at birth.

### Clinical characteristics

#### Concurrent mental health treatment

Concurrent treatment was defined as mental health treatment at any point in the study. At baseline, participants were asked to indicate if they were currently seeing a therapist for mental health concerns and/or if they were taking medications for a psychiatric condition. At the Week 8 assessment, participants were asked if psychotropic medications and/or psychotherapy were continued or if either was started during the course of the 8-week study intervention.

#### Patient Health Questionnaire (PHQ-8)

The PHQ-8 is a widely used 8-item self-report measure, with demonstrated reliability and sensitivity to clinical change. It is used to screen for depression, measure symptom severity, and assess outcome [41]. Respondents are asked to consider symptoms over the past two weeks and to indicate frequency of symptoms from not at all (0) to nearly every day (3). The total sum scores range from 0–24, with the following severity cutoffs—minimal (0–4), mild (5–9), moderate (10–14), moderately severe (15–19), and severe (20–24). A score of 10 or greater is considered an acceptable clinical cutoff for current depression [41]. PHQ-8 scores at baseline and Week 8 were analyzed in this study.

#### Generalized Anxiety Disorder (GAD-7)

The GAD-7 is a brief self-report tool to assess the frequency and severity of anxious thoughts and behaviors [42]. Respondents are asked to indicate frequency of symptoms from not at all (0) to nearly every day (3) over the previous two weeks. Total sum scores range from 0–21, with the following severity cutoffs—minimal (0–4), mild (5–9), moderate (10–14), and severe (15–21). A score of 10 or greater is considered a reasonable clinical cutoff for generalized anxiety disorder [42].

### W-MA-02 utilization

Over the course of the study, we tracked participant W-MA-02 log-ins. Completion of the.

intervention was defined as logging in to W-MA-02 during at least 50% (i.e., at least 4 of 8) of the study weeks (yes vs. no).

### Statistical analysis

Each set of analyses was performed on the two analytic subsamples: study participants with PHQ-8 ≥ 10 at baseline and those with GAD-7 ≥ 10 at baseline. Participants with clinically elevated symptoms of both depressive and anxiety symptoms were included in both subsamples (although multiple regression models adjusted for the presence of the other symptoms in analyses). Continuous demographic and clinical variables were summarized by means and standard deviations; discrete and/or ordinal variables were summarized by reporting the sample size and proportion of participants in each category. Response was defined as having a 50% reduction in PHQ-8 measured depressive symptoms among those with PHQ-8 ≥ 10 at baseline and having a 50% reduction in GAD-7 measured anxiety symptoms among those with GAD-7 ≥ 10 at baseline.

To address the first research question, a paired t-test was applied to the baseline and Week 8 PHQ-8 scores for the subgroup defined as having a baseline PHQ-8 ≥ 10. Similarly, for those with elevated anxiety symptoms at baseline (GAD-7 ≥ 10), a paired t-test was applied to the baseline and Week 8 GAD-7 scores to address the second research question. For both instances, the tests were intent-to-treat and two-sided, with alpha = 0.05.

To assess the third research question, sets of bivariate and multiple linear regression models were separately fit to the subgroups with baseline PHQ-8 ≥ 10 and baseline GAD-7 ≥ 10. Each regression model included change in depressive or anxiety symptom scores as outcomes, respectively, and each demographic and clinical characteristic as independent variables. First, bivariate regressions were run between each outcome and each demographic and clinical characteristic, separately. Then, a multiple regression model that included all demographic and clinical characteristics as well as an indicator of utilization to adjust for W-MA-02 use (dichotomous variable of having any W-MA-02 use in at least 50%, or 4 of 8, weeks of the study) were run and, using Akakie’s Information Criterion (AIC) for model selection, rerun until the final model was attained. Final models also removed all variables with Variance Inflation Factors greater than 4 to account for multicollinearity. Each model was run on the ITT population with no accommodations for missing data (i.e., those with missing data were dropped from individual bivariate or multiple regression models in each instance). Sensitivity analyses were performed with the PP population, which included participants who used the app in at least 4 of 8 study weeks and who completed the end of study (EOS) PHQ-8 and GAD-7 assessments; the same modeling approach used for the intention to treat (ITT) population was applied to the PP population. The level of significance for all final models was 0.05. All analyses were performed with the R statistical software program [43].

## Results

### Sample

**Fig. 3:**
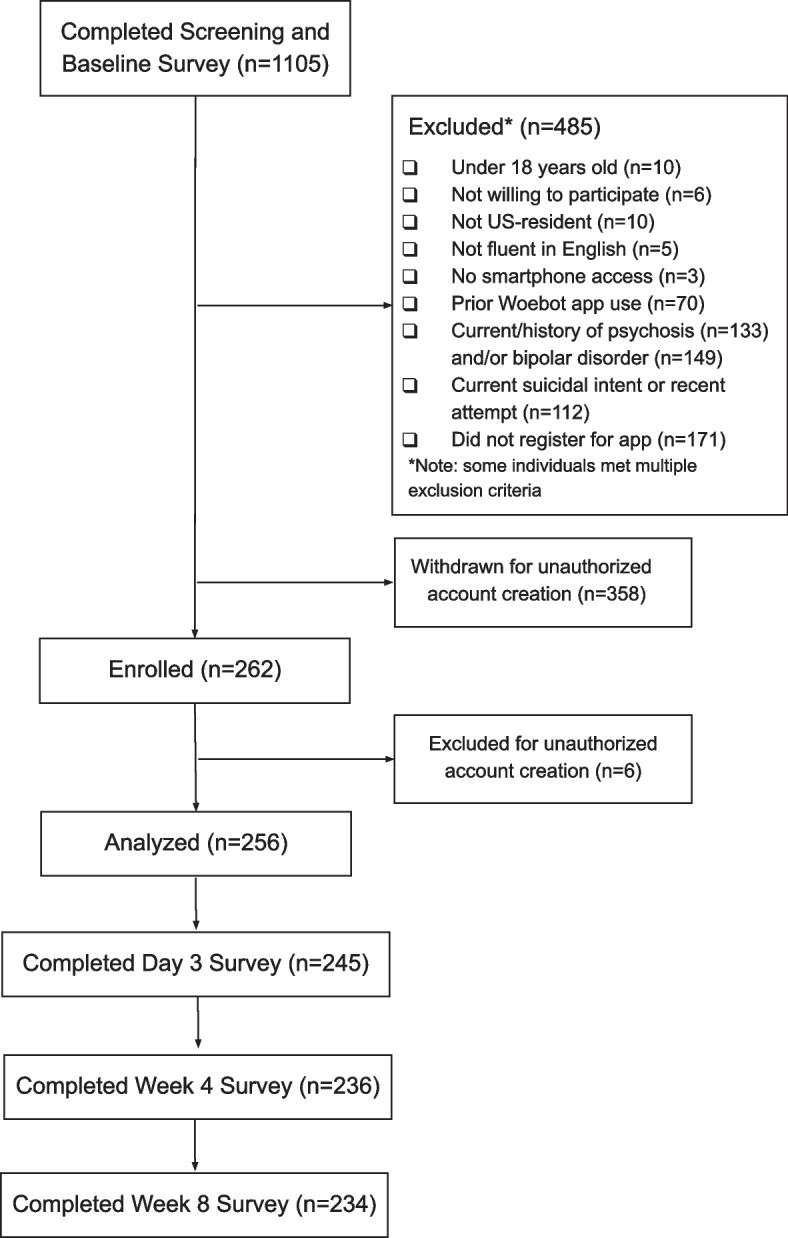
CONSORT diagram. Although Day 3 and Week 4 survey data were collected as part of the parent study, they were not part of the analyses included in the present report. Six participants were removed after enrollment due to a clerical error; they should have been removed prior to enrollment

As detailed in the CONSORT diagram (Fig. 3), 1105 potential participants were screened. Of these, 485 were excluded for not meeting eligibility criteria. After screening and baseline assessments were conducted, an additional 358 participants with unauthorized accounts (e.g., duplicate registrants) were withdrawn from the study and excluded from the analyses. Of the original 262 enrollees, an additional six unauthorized registrants began to complete study procedures but were subsequently removed from the analytic sample because they were initially missed as a result of a clerical error. Thus, the final analytic sample consisted of 256 participants. Of these 256 participants, 245 completed the Day 3 survey, 236 completed the Week 4 surveys, and 234 completed the Week 8 surveys.

### Sample characteristics

The analytic subsamples included 111 participants with elevated baseline levels of depressive symptoms and 107 participants with elevated baseline levels of anxiety symptoms at baseline as defined by a PHQ-8 score ≥ 10 and/or GAD-7 score ≥ 10, respectively. The sample size for the combined (elevated on PHQ-8 or GAD-7) groups comprised 139 participants; some participants with elevated symptoms of both depressive and anxiety symptoms were in both analytic subsamples (*n* = 79). Both the depressive symptom and anxiety symptom samples were predominantly female, educated, and employed, identified as non-Hispanic white and heterosexual, and had private health insurance (Table 1) with mean ages of 38 and 37 years, respectively. Among those with clinical levels of depressive symptoms, 50% had symptoms falling in the moderately severe or severe range (PHQ-8 score ≥ 15) and 49% reported concurrent mental health treatment during the study. Among those with clinical levels of anxiety symptoms, 44% had symptoms falling in the severe range (GAD-7 score ≥ 15) and 53% reported concurrent mental health treatment during the study.

**Table 1:** Baseline sociodemographic and clinical characteristics, adherence, and satisfaction among participants with clinical levels of baseline depressive or anxiety symptoms

	Participants with PHQ-8 baseline score ≥ 10 *N* = 111 n (%)	Participants with GAD-7 baseline score ≥ 10 *N* = 107 n (%)
**Sociodemographic Characteristic**		
Age in years (mean, sd)	38 (13.57)	37 (12.28)
Race/ethnicity		
Non-Hispanic Black	37 (33%)	32 (30%)
Non-Hispanic White	55 (50%)	56 (52%)
Other	19 (17%)	19 (18%)
Sex at Birth		
Female	83 (75%)	81 (76%)
Male	28 (25%)	26 (24%)
Sexual Orientation		
Sexual Minority	19 (17%)	19 (18%)
Heterosexual	92 (83%)	88 (82%)
Education		
Graduate or Postgraduate Degree	32 (29%)	24 (23%)
College Degree	43 (39%)	43 (41%)
Some College or technical school	21 (19%)	22 (21%)
High School (Grades 9—12)	14 (13%)	16 (15%)
Employment		
Full Time Employed	53 (49%)	57 (54%)
Part Time Employed	11 (10%)	9 (9%)
Not Employed	29 (27%)	22 (21%)
Other	15 (14%)	17 (16%)
Marital Status		
Divorced/Separated/Widowed	10 (9%)	10 (10%)
Married/Partnered/cohabiting	50 (47%)	48 (46%)
Single	47 (44%)	47 (45%)
Health Insurance		
Government Based Insurance	40 (37%)	37 (36%)
Private Insurance	46 (43%)	45 (43%)
No Insurance/ Prefer not to answer	21 (20%)	22 (21%)
**Clinical Characteristics**		
Baseline Depressive symptom severity		
Minimal	0 (0%)	7 (7%)
Mild	0 (0%)	21 (20%)
Moderate	55 (50%)	25 (23%)
Moderate-severe	30 (27%)	29 (27%)
Severe	26 (23%)	25 (23%)
Baseline Anxiety symptom severity		
Minimal	3 (3%)	0 (0%)
Mild	29 (26%)	0 (0%)
Moderate	37 (33%)	60 (56%)
Severe	42 (38%)	47 (44%)
Concurrent Mental Health Treatment^a^		
Any (Concurrent treatment)	54 (49%)	57 (53%)
None (Woebot only)	57 (51%)	50 (47%)
**Adherence Metrics**		
App Utilization		
Use of W-MA-02 in at least 4 of 8 weeks	74 (67%)	69 (64%)
Per protocol data set	71 (64%)	63 (59%)

“Per protocol” was defined as using W-MA-02 in at least 4 of 8 study weeks and completing end of study PHQ-8 and GAD-7 assessments

*GAD-7* Generalized Anxiety Disorder-7 item scale, *PHQ-8* Patient Health Questionnaire-8 item scale

^a^Concurrent mental health treatment = any psychotherapy or psychotropic medication use at any time during the study

#### Per Protocol (PP) characteristics

The assessment completion rate at Week 8 was 91%. The PP population examined in sensitivity analyses comprised 177 (69%) of the 256 participants in the analytic sample after removing 67 (26%) who used W-MA-02 for at most 3 of 8 weeks and removing 22 (9%) whose EOS PHQ-8 and GAD-7 assessments at Week 8 were fully missing (no item scores were completed for each measure); the removals were not mutually exclusive (*n* = 10 met both criteria). Sixty-four percent (*N* = 71/111) of the elevated depressive symptoms subgroup met PP and 59% (*N* = 63/107) of the elevated anxiety symptoms subgroup met these criteria.

### Adverse events

No study participant reported an AE or serious adverse event (SAE) during the study. Over the course of the study, 127,773 messages were sent by the 256 participants. A subset of 71 participants provided text inputs containing language suggestive of a potential crisis. Of these participants, four confirmed being in a crisis situation and were automatically provided resources and support as described in the Ethical Considerations section of the Methods.

### Depressive symptoms

In the subgroup with clinically elevated depressive symptoms, PHQ-8 scores significantly decreased over the 8-week study period (mean change -7.28, SD = 5.91, Cohen’s d = -1.23, *p* < 0.001); 43% responded to the intervention. In bivariate regression models, the amount of symptom decline did not significantly differ by age, educational level, employment, or baseline level of anxiety symptoms but did significantly differ across several characteristics tested (Table 2). Non-Hispanic Blacks, those who were single, and those with severe levels of baseline depressive symptoms demonstrated significantly greater amounts of decline in depressive symptoms than Non-Hispanic Whites, those who were married/partnered/cohabiting, and those with moderate levels of baseline depressive symptoms, respectively. Females, participants identifying as sexual minorities, those with private or government-based health insurance, those who used the program on at least 4 of the 8 weeks, and those in concurrent mental health treatment demonstrated significantly lower amounts of decline in depressive symptoms than males, participants identifying as heterosexuals, those with no health insurance or who declined to answer the health insurance question, those who used the program on less than 4 of the 8 weeks, and those not in concurrent mental health treatment, respectively. After removing race/ethnicity, insurance, and employment from consideration because of multicollinearity, the final multiple regression model indicated significant differences in the amount of decrease in depressive symptoms at Week 8 by sexual orientation, marital status, baseline depressive symptom severity category, and concurrent mental health treatment (Table 3) in the same directions as each bivariate model described above (in addition, divorced individuals had greater amounts of depressive symptom declines as compared with married/partnered, cohabiting). Although a few bivariate analyses had varying levels of significance across ITT and PP analyses (Supplemental Table 1), the multiple regression model largely confirmed the full sample findings (Supplemental Table 2).

**Table 2:** Unadjusted bivariate linear regression models of characteristics associated with change scores from baseline to Week 8 in depressive symptoms among those with clinically elevated levels of baseline depressive symptoms: PHQ-8 ≥ 10

*Characteristics of Interest*	**PHQ-8: Week 8 Change Scores**		
	*Estimates*	*95% CI*	*p-value*
Use of W-MA-02 in at least 4 of 8 weeks	2.90	0.51, 5.40	**0.02**
Age	0.03	-0.05, 0.12	0.40
Race/Ethnicity			
Non-Hispanic White	Reference Level		
Non-Hispanic Black	-6.00	-8.30, -3.70	**< 0.01**
Other	0.02	-2.80, 2.90	> 0.90
Sex at Birth			
Male	Reference Level		
Female	2.70	0.09, 5.30	**0.04**
Sexual Orientation:			
Heterosexual	Reference Level		
Sexual Minority	5.60	2.60, 8.60	**< 0.01**
Education			
High School	Reference Level		
College Degree	-1.90	-5.60, 1.80	0.30
Graduate or postgraduate degree	-0.68	-4.40, 3.10	0.70
Some college or technical school	1.10	-3.10, 5.30	0.60
Employment			
Full Time	Reference Level		
Not Employed	2.40	-0.36, 5.20	0.09
Other	3.10	-0.49, 6.80	0.09
Part Time Employed	3.60	-0.32, 7.40	0.07
Marital Status			
Married/Partnered/Cohabiting	Reference Level		
Divorced/Separated/Widowed	-2.20	-6.20, 1.70	0.30
Single	-4.10	-6.60, -1.70	**< 0.01**
Health Insurance:			
No Insurance/Prefer not to answer	Reference Level		
Government based Insurance	6.90	4.20, 9.70	**< 0.01**
Private Insurance	7.50	4.80, 10.00	**< 0.01**
BL Depressive Symptom Severity			
Moderate	Reference Level		
Moderate-Severe	-1.10	-3.20, 1.00	0.30
Severe	-9.30	-12.00, -7.00	**< 0.01**
BL Anxiety Symptom Severity			
Minimal	Reference Level		
Mild	-1.60	-9.50, 6.20	0.70
Moderate	-2.10	-9.90, 5.70	0.60
Severe	-7.10	-15.00, 0.71	0.07
Concurrent Mental Health Treatment^a^	3.20	0.94, 5.40	< **0.01**

*BL* baseline, *PHQ-8* Patient Health Questionnaire-8 item scale

^a^ Concurrent mental health treatment = any psychotherapy or psychotropic medication use at any time during the study

**Table 3:** Adjusted linear regression model (Multiple Regression) of change scores from baseline to Week 8 in depressive symptoms among those with clinically elevated levels of baseline depressive symptoms: PHQ-8 ≥ 10

*Characteristics of Interest*	**PHQ-8: Week 8 Change Scores**		
	*Estimates*	*95% CI*	*p-value*
(Intercept)	-2.46	-10.16 – 5.24	0.53
Used the app in at least 4 of 8 weeks	0.20	-1.75 – 2.15	0.84
Age	0.05	-0.03 – 0.14	0.23
Sex at Birth			
Male	Reference Level		
Female	-0.96	-3.24 – 1.32	0.41
Sexual Orientation			
Heterosexual	Reference Level		
Sexual Minority	5.71	2.88 – 8.54	**< 0.01**
Education			
High School	Reference Level		
College Degree	-1.63	-4.33 – 1.07	0.23
Graduate or postgraduate degree	-0.24	-3.18 – 2.70	0.87
Some college or technical school	0.88	-2.26 – 4.01	0.58
Marital Status			
Married/Partnered/Cohabiting	Reference Level		
Divorced/Separated/Widowed	-3.86	-7.38 – -0.34	**0.03**
Single	-3.75	-5.78 – -1.72	**< 0.01**
BL Depressive Symptom Severity			
Moderate	Reference Level		
Moderate-Severe	-0.09	-2.44 – 2.25	0.94
Severe	-5.32	-8.16 – -2.47	**< 0.01**
BL Anxiety Symptom Severity			
Minimal	Reference Level		
Mild	-3.97	-10.29 – 2.35	0.215
Moderate	-4.50	-11.02 – 2.02	0.17
Severe	-5.59	-12.17 – 0.99	< 0.01
Concurrent Mental Health Treatment^a^	2.62	0.80 – 4.43	< **0.01**

Final models did not consider race/ethnicity, employment, or insurance status because of multicollinearity

*BL* baseline, *PHQ-8* Patient Health Questionnaire-8 item scale

^a^ Concurrent mental health treatment = any psychotherapy or psychotropic medication use at any time during the study

### Anxiety symptoms

Average GAD-7 scores for each subgroup of interest significantly decreased over the course of the study. In the subgroup with clinically elevated anxiety symptoms at baseline, GAD-7 scores significantly decreased over the 8-week study period (mean change -7.45, SD = 5.99, Cohen’s d = -1.24, *p* < 0.001); 58% responded to the intervention. The amount of symptom decline did not significantly differ by use of W-MA-02 at least 4 of 8 weeks, age, sex at birth, educational level, or employment, but did significantly differ across several characteristics tested (Table 4). Non-Hispanic Blacks, those who were single, those with severe levels of baseline depressive symptoms, and those with severe levels of anxiety symptoms demonstrated greater amounts of decline in anxiety symptoms than Non-Hispanic Whites, those who were married/partnered/cohabiting, those with minimal levels of baseline depressive symptoms, and those with moderate levels of baseline anxiety symptoms, respectively. Participants identifying as sexual minorities, those with private or government-based health insurance, and those in concurrent mental health treatment demonstrated lower amounts of decline in anxiety symptoms than heterosexuals, those with no health insurance or who declined to answer the health insurance question, and those without concurrent mental health treatment, respectively. PP analyses largely confirmed the full sample findings, with the exception of concurrent mental health treatment reaching marginal significance (*p* = 0.05, Supplementary Table 3).

**Table 4:** Unadjusted bivariate linear regression models of change scores from baseline to Week 8 in anxiety symptoms among those with clinically elevated levels of baseline anxiety symptoms: GAD-7 ≥ 10

*Characteristics of Interest*	**GAD-7: Week 8 Change Scores**		
	*Estimates*	*95% CI*	*p-value*
Use of W-MA-02 in at least 4 of 8 weeks	2.20	-0.25, 4.70	0.08
Age	0.03	-0.07, 0.14	0.50
Race/Ethnicity			
Non-Hispanic White	Reference Level		
Non-Hispanic Black	-5.7	-8.20, -3.20	**< 0.01**
Other	-0.12	-3.00, 2.80	> 0.90
Sex at Birth			
Male	Reference Level		
Female	1.80	-1.00, 4.60	0.20
Sexual Orientation			
Heterosexual	Reference Level		
Sexual Minority	3.70	0.65, 6.80	**0.02**
Education			
High School	Reference Level		
College Degree	-1.70	-5.30, 1.90	0.30
Graduate or postgraduate degree	-1.80	-5.70, 2.10	0.40
Some college or technical school	-0.28	-4.50, 3.90	0.90
Employment			
Full Time	Reference Level		
Not Employed	2.10	-1.00, 5.20	0.20
Other	1.20	-2.20, 4.70	0.50
Part Time Employed	2.30	-2.2, 6.8	0.3
Marital Status			
Married/Partnered/Cohabiting	Reference Level		
Divorced/Separated/Widowed	-1.50	-5.80, 2.80	0.50
Single	-2.90	-5.40, -0.37	**0.03**
Health Insurance			
No Insurance/Prefer not to answer	Reference Level		
Government based Insurance	5.10	2.10, 8.00	**< 0.01**
Private Insurance	6.80	3.90, 9.60	**< 0.01**
BL Depressive Symptom Severity			
Minimal	Reference Level		
Mild	-1.40	-6.20, 3.40	0.60
Moderate	-0.71	-5.30, 3.90	0.80
Moderate-Severe	-0.12	-4.60, 4.40	> 0.90
Severe	-7.3	-12.00, -2.70	< **0.01**
BL Anxiety Symptom Severity			
Moderate	Reference Level		
Severe	-4.90	-7.10, -2.70	**< 0.01**
Concurrent Mental Health Treatment^a^	3.20	0.90, 5.60	< 0.01

*BL* baseline, *GAD-7* Generalized Anxiety Disorder-7 item scale

^a^ Concurrent mental health treatment = any psychotherapy or psychotropic medication use at any time during the study

After removing race/ethnicity, employment, and insurance status from consideration due to multicollinearity, the final multiple regression model indicated significant differences in the amount of decrease in anxiety symptoms at Week 8 by sexual orientation, marital status, baseline anxiety symptom severity category, and concurrent mental health treatment (Table 5) in the same direction as those previously described in each bivariate regression model. PP analyses largely confirmed the full sample findings (Supplemental Tables 3 and 4).

**Table 5:** Adjusted Linear Regression Model (Multiple Regression) of change scores from baseline to Week 8 in anxiety symptoms among those with clinically elevated levels of baseline anxiety symptoms: GAD-7 ≥ 10

*Characteristics of Interest*	**GAD-7: Week 8 Change Scores**		
	*Estimates*	*95% CI*	*p-value*
(Intercept)	-4.68	-10.40 – 1.03	0.11
Used the app in at least 4 of 8 weeks	1.26	-1.06 – 3.59	0.28
Age	0	-0.11 – 0.11	0.98
Sex at Birth:			
Male	Reference Level		
Female	-1.76	-4.63 – 1.10	0.22
Sexual Orientation:			
Heterosexual	Reference Level		
Sexual Minority	4.47	1.20 – 7.74	**0.01**
Education Level:			
High School	Reference Level		
College Degree	-1.7	-4.79 – 1.39	0.28
Graduate or postgraduate degree	-1.12	-4.72 – 2.48	0.54
Some college or technical school	-0.55	-4.38 – 3.28	0.78
Marital Status:			
Married/Partnered/Cohabiting	Reference Level		
Divorced/Separated/Widowed	-2.85	-7.30 – 1.61	0.21
Single	-4.19	-6.69 – -1.68	< **0.01**
BL Anxiety Symptom Severity:			
Moderate	Reference Level		
Severe	-3.88	-6.14 – -1.62	< **0.01**
Concurrent Mental Health Treatment^a^	3.2	0.95 – 5.45	< **0.01**

Final models did not consider race/ethnicity, employment, and insurance status due to multicollinearity

*BL* baseline, *GAD-7* Generalized Anxiety Disorder-7 item scale

^a^ Concurrent mental health treatment = any psychotherapy or psychotropic medication use at any time during the study

## Discussion

In this single-arm exploratory trial of W-MA-02, an intervention employing an NLP-supported relational agent, the objective was to explore the magnitude of the reduction in self-reported depressive (PHQ-8) and anxiety (GAD-7) symptoms between baseline and Week 8 EOI as well as the associative relationships between demographic and clinical characteristics and each outcome, among those that had elevated scores on these measures at baseline. On average, study participants with elevated depressive symptoms at baseline experienced significant declines in self-reported depressive symptoms across the intervention period; study participants with elevated anxiety symptoms at baseline experienced significant declines in self-reported anxiety symptoms across the intervention period. In addition, analyses revealed significant associations between depressive and anxiety symptom changes and demographic and clinical characteristics in both bivariate and, in many cases, multiple regression models. The high assessment completion and W-MA-02 utilization rates are notable given that attrition remains a perennial problem in mobile health app studies [44], with one recent systematic review of depression DMHI app RCTs suggesting that dropout rates may range from 26 to 48%, even when participant reimbursement was included in the analysis [45].

In the absence of a control group, it is difficult to draw conclusions about the efficacy of W-MA-02, especially given evidence that approximately one-third of untreated major depression or generalized anxiety disorder cases may spontaneously remit within 6 months [46–48]. However, in both the clinically elevated depressive and anxiety groups, we noted significant decreases in self-reported symptoms at Week 8. The mean level of change was greater than seven points on both change in PHQ-8 among those with clinically elevated depressive symptoms at baseline as well as change in GAD-7 among those with clinically elevated anxiety symptoms at baseline. This is noteworthy given that these outcomes were two and three points greater, respectively, than the typically accepted change score cutoff of five and four points to define a clinically significant response on the PHQ-8 and GAD-7 measures, respectively [49, 50].

Our adjusted analyses did not find significant associations between sex at birth, age, educational background, employment, and clinical outcomes, which is consistent with prior psychotherapeutic and DMHI meta-analyses and systematic reviews [8, 51–53]. However, several studies of internet-based CBT (iCBT) have found better depression outcomes for females [26, 54] and greater probability of non-response in anxiety disorders and depression for males [55]. Our results may support the finding that age and sex at birth may be more important to the epidemiology of depression than to prognosis with an intervention [53].

The associations that were found in adjusted analyses between significant improvements in depression and anxiety symptoms and sexual orientation, concurrent mental health treatment, marital status, and baseline symptom severity suggest that these may be demographic predictors of intervention response to DMHIs employing relational agents. However, a series of purpose-designed follow-up studies to test each of these putative predictors of clinical outcome in a randomized clinical trial (RCT) design is needed before any definitive conclusions can be made. Given that LGBTQ individuals have demonstrated higher rates of depression and anxiety [56] that are further compounded by access barriers and stigma [31], DMHIs may present an unique opportunity to provide safe and accessible support to this group. LGBTQ individuals are also more likely to seek health information with digital resources than their non-LGBTQ counterparts [57] and a high proportion of people accessing digital text-based support services identify as belonging to sexual and gender minorities [58]. Thus, further studies among this group should be a research priority. Finally, our study noted significant differences in the association of concurrent care involvement with changes in anxiety and depression symptoms, but this concurrent care was received independently and therefore no details on its characteristics (e.g., description of intervention, frequency, fidelity to psychotherapeutic treatment model, modality of delivery, dose, and route of psychopharmacological agents) were available. Future studies should test if this association holds in a study using a prospective, RCT design as well as in a true blended care model that tightly integrates the relational-agent-delivered DMHI with human support.

Further, historical findings regarding the link between marital status and psychotherapy outcomes have been decidedly mixed [59], but in this study we found that single or divorced/separated/widowed individuals in the elevated depression group and single individuals in the elevated anxiety group experienced more improved outcomes compared to their married/partnered/cohabiting peers in adjusted analyses. These results are somewhat consistent with a study of an online cognitive behavior intervention compared to a waiting list control that demonstrated greater improvements in depression outcomes at 4 months among separated, widowed, or divorced individuals as compared to married participants [60]. Due to the importance of social support in mental health treatment [51], a more detailed assessment of factors such as relationship quality may better explain the relationship between marital status and clinical outcomes [51].

Finally, we found that greater baseline depression and anxiety symptom severity was associated with greater symptom improvements in the elevated depression and anxiety groups, respectively. It is notable that 50% of the elevated depression group reported either moderately severe or severe levels of baseline symptoms and 44% of the elevated anxiety groups reported severe symptoms. DMHIs have often been considered as interventions for mild to moderate symptoms [61], but these findings may suggest promise for populations with a broader range of severity. While it is true that those with elevated symptoms have more room to improve [8], we nonetheless believe that this finding suggests that there may be clinical utility for programs like this beyond mild to moderate severity levels with adequate safety checks in place.

### Strengths and limitations

The present study adds to the extant mental health outcomes literature by extending the investigation of important demographic and clinical characteristics in DMHI interventions to one delivered specifically via a relational agent. To our knowledge, this is the first study that has been expressly designed for this purpose. The recruitment of a demographically diverse naturalistic sample allowed a broad assessment of characteristics that have been traditionally underexplored in digital health studies, such as racial/ethnic membership, sexual orientation, employment status, and health insurance status. There has been increasing attention to the lack of diversity in marginalized and underserved participants involved in DMHI studies [31], and this study notably recruited much higher proportions of sexual minorities [62] and non-Hispanic Black [63] individuals than are found in the U.S. population. Despite a generally demographically diverse sample, it should be noted that this sample was about 75% female sex at birth, a proportion that is not unusual for health behavior studies [64]. Greater recruitment based on gender identity and participation of males would improve sample diversity in future studies to allow for greater generalization of findings.

Several limitations must be noted. First, the study was not an RCT and did not include a control group, so there may have been additional confounders or other factors affecting the efficacy of the intervention. For example, researchers have identified user expectancies related to digital device use (a.k.a.“digital placebo effect”) that may affect mental health outcomes [65]. Subgroup differences, some of which had small sample sizes so limited power to detect such differences, observed in the present study are preliminary and should ideally be tested in RCTs with larger sample sizes and a priori power calculations. Second, because the intervention was delivered over an 8-week period, the longer-term durability of any effects is not known. Changes that did occur might be explained by measurement reactivity, as small effects have been observed in health behavior change [66] and digital health studies [67]. Symptom improvements may also be explained in part by spontaneous remission [46, 47, 68] or other participant characteristics that were not assessed as part of the study. Third, symptoms and concurrent treatments were assessed via self-report and there was no independent confirmation of participants' clinical presentation or therapeutic involvements. Although about half of the sample reported receiving concurrent treatment (consistent with national findings that only about half of those in need receive mental health care [69]), we do not know the nature, duration, or intensity of psychotherapies or psychotropic medications received or if participants engaged in other interventions (i.e., not delivered by a therapist or receipt of medication) In addition, although we collected information about both psychotherapy and psychotropic medications separately, the sample size was not sufficient to make comparisons between these modalities and so they were pooled for analysis. Future studies would ideally collect detailed information about the psychotherapies being received, as well as full details about concurrent psychotropic medications. Fourth, studies of online interventions can be affected by certain types of data quality issues (e.g. fraudulent participants, distraction, inattention, or intoxication during use) that may benefit from inclusion of quality checks in future experimental design [70, 71]. Fifth, although demographically diverse for a naturalistic sample, the study population was nonetheless majority college or advanced degree holders and employed. Additional studies targeting the mental health needs of those with lower levels of educational attainment and/or who may be facing under- or unemployment are needed. Finally, our definition of “per protocol” as using W-MA-02 in at least 4 of 8 study weeks and completing EOS depressive and anxiety symptom assessments may not have been granular enough to account fully for achieved outcomes or to reveal unique patterns of disengagement. Exploring alternative engagement metrics would enhance our understanding of relational agent usage and the relationship of outcomes to user characteristics.

## Conclusions

The present study suggests early promise for a DMHI that employs an easily scalable NLP-based relational agent incorporating elements of CBT, DBT, and IPT to a demographically diverse naturalistic sample experiencing self-reported symptoms of depression and/or anxiety. Although exploratory in nature, this study reveals intriguing areas for further research that can help optimize the unique role DMHIs that include relational agents can play in filling a mental health service gap that has only widened in the wake of the COVID-19 pandemic.

### Supplementary Information

**Additional file 1.**

## Data Availability

The datasets underlying the results of this study are confidential and include proprietary commercial information. Requests can be submitted to the corresponding author and will be subject to review and approval.

## Abbreviations

ACT: Acceptance and commitment therapy
AE/SAE: Adverse and serious adverse events
CBT: Cognitive behavioral therapy
DASS-21: Depression Anxiety Stress Scale-21
DBT: Dialectical Behavior Therapy
DMHI: Digital Mental Health Intervention
EDC: Electronic Data Capture
EOI: End of intervention
EOS: End of study
GAD-7: Generalized Anxiety Disorder
iCBT: Internet-based cognitive behavior therapy
IPT: Interpersonal Psychotherapy
ITT: Intention to treat
LGBTQ: Lesbian, gay, bisexual, transgender, queer or questioning
MBCT: Mindfulness-based cognitive therapy
NLP: Natural language processing
PHQ-8: Patient Health Questionnaire
PP: Per protocol
RCT: Randomized controlled trials
U.S.: United States

## Declarations

### DIsclaimer

Woebot for Mood & Anxiety (W-MA-02) is an investigational medical device. It has not been evaluated, cleared, or approved by the FDA.
