# Supplementary material for: Demographic and clinical characteristics associated with anxiety and depressive symptom outcomes in users of a digital mental health intervention incorporating a relational agent
Source: BMC Psychiatry. 2024 Jan 30;24:79. doi: 10.1186/s12888-024-05532-6 (PMC10826101; doi:10.1186/s12888-024-05532-6)
Supplement: Supplementary file 1 — Additional file 1. [file 12888_2024_5532_MOESM1_ESM.docx]

**Supplementary Table 1. Unadjusted Bivariate Linear Regression Models of Characteristics Associated with change scores from baseline to Week 8 in depressive symptoms among those with clinically elevated levels of baseline depressive symptoms: PHQ-8 ≥ 10: Per Protocol Analysis**

|  | **PHQ-8: Week 8 Change Scores** | | |
| --- | --- | --- | --- |
| *Characteristics of Interest* | *Estimates* | *95% CI* | *p-value* |
| Age | 0.03 | -0.05, 0.12 | 0.40 |
| Race/Ethnicity  Non-Hispanic White | Reference Level | | |
| Non-Hispanic Black | -4.00 | -6.70, -1.40 | **<0.01** |
| Other | -0.09 | -3.30, 3.10 | >0.90 |
| Sex at Birth  Male | Reference Level | | |
| Female | 3.50 | 0.65, 6.40 | **0.02** |
| Sexual Orientation:  Heterosexual | Reference Level | | |
| Sexual Minority | 5.20 | 1.90, 8.50 | **<0.01** |
| Education  High School | Reference Level | | |
| College Degree | 0.64 | -3.40, 4.70 | 0.80 |
| Graduate or postgraduate degree | 1.90 | -2.30, 6.00 | 0.40 |
| Some college or technical school | 1.40 | -3.10, 5.90 | 0.50 |
| Employment  Full Time | Reference Level | | |
| Not Employed | -0.23 | -3.10, 2.70 | 0.90 |
| Other | -0.11 | -4.50, 4.30 | >0.90 |
| Part Time Employed | 1.80 | -2.30, 6.00 | 0.40 |
| Marital Status  Married/Partnered/Cohabiting | Reference Level | | |
| Divorced/Separated/Widowed | -2.50 | -6.60, 1.60 | 0.20 |
| Single | -4.00 | -6.60, -1.30 | **<0.01** |
| Health Insurance:  No Insurance/Prefer not to answer | Reference Level | | |
| Government based Insurance | 2.60 | -0.66, 5.90 | 0.11 |
| Private Insurance | 3.30 | -0.08, 6.70 | 0.06 |
| BL Depressive Symptom Severity  Moderate | Reference Level | | |
| Moderate-Severe | -1.00 | -3.50 1.50 | 0.40 |
| Severe | -6.80 | -9.70, -4.00 | **<0.01** |
| BL Anxiety Symptom Severity  Minimal | Reference Level | | |
| Mild | -1.50 | -8.90, 5.90 | 0.70 |
| Moderate | -2.60 | -10.00, 4.80 | 0.50 |
| Severe | -4.80 | -12.00, 2.60 | 0.20 |
| Concurrent Mental Health Treatment* | 1.80 | -0.66, 4.20 | 0.20 |

BL = baseline; PHQ-8 = Patient Health Questionnaire-8 item scale

* Concurrent mental health treatment = any psychotherapy or psychotropic medication use at any time during the study

NOTE: Per protocol defined as using the app in at least 4 of the 8 study weeks and completing the end of study PHQ-8 and GAD-7 assessments.

**Supplemental Table 2. Adjusted Linear Regression Model (Multiple Regression) of change scores from baseline to Week 8 in depressive symptoms among those with clinically elevated levels of baseline depressive symptoms: PHQ-8 ≥ 10: Per Protocol Analysis**

|  | **PHQ-8: Week 8 Change Scores** | | |
| --- | --- | --- | --- |
| *Characteristics of Interest* | *Estimates* | *95% CI* | *p-value* |
| (Intercept) | -8.12 | -12.53 – -3.70 | **<0.01** |
| Age | 0.06 | -0.02 – 0.15 | 0.15 |
| Sex at Birth  Male | Reference Level | | |
| Female | -0.28 | -3.14 – 2.57 | 0.84 |
| Sexual Orientation  Heterosexual | Reference Level | | |
| Sexual Minority | 5.37 | 2.23 – 8.50 | **<0.01** |
| Marital Status  Married/Partnered/Cohabiting | Reference Level | | |
| Divorced/Separated/Widowed | -3.38 | -7.05 – 0.29 | 0.07 |
| Single | -4.06 | -6.43 – -1.69 | **<0.01** |
| BL Depressive Symptom Severity  Moderate | Reference Level | | |
| Moderate-Severe | -0.20 | -2.66 – 2.25 | 0.87 |
| Severe | -4.89 | -7.71 – -2.07 | **<0.01** |
| Concurrent Mental Health Treatment* | 2.37 | 0.28 – 4.46 | **0.03** |

BL = baseline; PHQ-8 = Patient Health Questionnaire-8 item scale

* Concurrent mental health treatment = any psychotherapy or psychotropic medication use at any time during the study

NOTE: Final models did not consider race/ethnicity, employment, or insurance status because of multicollinearity. Per protocol defined as using the app in at least 4 of the 8 study weeks and completing the end of study PHQ-8 and GAD-7 assessments.

**Supplemental Table 3: Unadjusted Bivariate Linear Regression Models of change scores from baseline to Week 8 in anxiety symptoms among those with clinically elevated levels of baseline anxiety symptoms: GAD-7 ≥ 10: Per Protocol Analysis**

|  | **GAD-7: Week 8 Change Scores** | | |
| --- | --- | --- | --- |
| *Characteristics of Interest* | *Estimates* | *95% CI* | *p-value* |
| Age | 0.06 | -0.05, 0.17 | 0.30 |
| Race/Ethnicity  Non-Hispanic White | Reference Level | | |
| Non-Hispanic Black | -3.90 | -6.80, -1.00 | **<0.01** |
| Other | -0.74 | -4.40, 2.90 | 0.70 |
| Sex at Birth  Male | Reference Level | | |
| Female | 3.20 | 0.01, 6.30 | **<0.05** |
| Sexual Orientation  Heterosexual | Reference Level | | |
| Sexual Minority | 3.20 | -0.35, 6.60 | 0.08 |
| Education  High School | Reference Level | | |
| College Degree | 1.20 | -2.70, 5.10 | 0.50 |
| Graduate or postgraduate degree | 1.60 | -2.70, 5.90 | 0.50 |
| Some college or technical school | 0.88 | -3.70, 5.50 | 0.70 |
| Employment  Full Time | Reference Level | | |
| Not Employed | 0.36 | -2.80, 3.50 | 0.80 |
| Other | -2.60 | -6.60, 1.30 | 0.20 |
| Part Time Employed | 1.80 | -2.90, 6.40 | 0.40 |
| Marital Status  Married/Partnered/Cohabiting | Reference Level | | |
| Divorced/Separated/Widowed | 0.00 | -4.50, 4.50 | >0.90 |
| Single | -3.00 | -5.70, -0.21 | **0.04** |
| Health Insurance  No Insurance/Prefer not to answer | Reference Level | | |
| Government based Insurance | 2.10 | -1.30, 5.60 | 0.20 |
| Private Insurance | 4.10 | 0.59, 7.70 | **0.02** |
| BL Depressive Symptom Severity  Minimal | Reference Level | | |
| Mild | 0.58 | -4.90, 6.10 | 0.80 |
| Moderate | 0.34 | -4.90, 5.60 | 0.90 |
| Moderate-Severe | 0.79 | -4.40, 5.90 | 0.80 |
| Severe | -3.20 | -8.60, 2.20 | 0.20 |
| BL Anxiety Symptom Severity  Moderate | Reference Level | | |
| Severe | -2.60 | -5.20, 0.04 | 0.05 |
| Concurrent Mental Health Treatment* | 2.60 | -0.02, 5.10 | 0.05 |

BL = baseline; GAD-7 = Generalized Anxiety DIsorder-7 item scale

* Concurrent mental health treatment = any psychotherapy or psychotropic medication use at any time during the study

NOTE: Per protocol defined as using the app in at least 4 of the 8 study weeks and completing the end of study PHQ-8 and GAD-7 assessments.

**Supplemental Table 4. Adjusted Linear Regression Model (Multiple Regression) of change scores from baseline to Week 8 in anxiety symptoms among those with clinically elevated levels of baseline anxiety symptoms: GAD-7 ≥ 10: Per Protocol Analysis**

|  | **GAD-7: Week 8 Change Scores** | | |
| --- | --- | --- | --- |
| *Characteristics of Interest* | *Estimates* | *95% CI* | *p-value* |
| (Intercept) | -8.67 | -15.80 – -1.54 | **0.02** |
| Age | 0.04 | -0.08 – 0.16 | 0.48 |
| Sex at Birth:  Male | Reference Level | | |
| Female | -0.14 | -3.67 – 3.38 | 0.94 |
| Sexual Orientation:  Heterosexual | Reference Level | | |
| Sexual Minority | 4.17 | 0.30 – 8.05 | **0.04** |
| Marital Status:  Married/Partnered/Cohabiting | Reference Level | | |
| Divorced/Separated/Widowed | -2.72 | -7.51 – 2.06 | 0.26 |
| Single | -3.81 | -6.81 – -0.81 | **0.01** |
| BL Anxiety Symptom Severity:  Moderate | Reference Level | | |
| Severe | -1.72 | -4.65 – 1.21 | 0.24 |
| BL Depressive Symptom Severity:  Minimal | Reference Level | | |
| Mild | 1.02 | -4.41 - 6.44 | 0.708 |
| Moderate | 0.07 | -4.96 - 5.10 | 0.98 |
| Moderate-Severe | 1.64 | -3.32 - 6.60 | 0.51 |
| Severe | -0.73 | -6.08 - 4.61 | 0.78 |
| Concurrent Mental Health Treatment* | 2.99 | 0.33 - 5.64 | **0.03** |

BL = baseline; GAD-7 = Generalized Anxiety DIsorder-7 item scale

* Concurrent mental health treatment = any psychotherapy or psychotropic medication use at any time during the study

NOTE: Final models did not consider race/ethnicity, employment, and insurance status due to multicollinearity. Per protocol defined as using the app in at least 4 of the 8 study weeks and completing the end of study PHQ-8 and GAD-7 assessments.
